# A gastrointestinal stromal tumor of stomach presenting with an intratumoral abscess: A case report

**DOI:** 10.1016/j.amsu.2021.01.091

**Published:** 2021-02-02

**Authors:** Ahmed Ballati, Zakaria Essaidi, Layla El Attar, Driss Errguibi, Amal Hajri, Rachid Boufettal, Saad Rifki El Jai, Farid Chehab

**Affiliations:** a Department of General Surgery, University Hospital Centre Ibn Rochd, Casablanca, Morocco; b Faculty of Medicine and Pharmacy, Hassan II University, Casablanca, Morocco

**Keywords:** Gastrointestinal stromal tumor, Intratumoral abscess, Stomach, Emergency surgery

## Abstract

**Introduction:**

Gastrointestinal stromal tumors (GISTs) are the most common mesenchymal tumors in the gastrointestinal tract, GISTs of the stomach presenting as an intratumoral abscess are extremely rare, which necessitates emergency surgery, we report a case of a stomach GIST developing an intratumoral abscess, in whom emergency surgery was performed.

**Presentation of case:**

A 68-year-old man presented with severe abdominal pain and a fever. Laboratory data showed an elevated white blood cell count and C-reactive protein level. Computed to mography scan showed a 15 × 10 cm cystic mass adjacent to greater curvature of the stomach, which contained air. Emergency laparotomy revealed A giant cystic gastric mass was observed. Sleeve gastrectomy were performed. Immunohistochemically, the tumor was diagnosed as a Gastric high risk GIST,and imatinib mesylate was initiated, The patient had an uneventful postoperative course and remains well.

**Discussion and conclusion:**

Such rare cases can be diagnosed and treated properly with careful clinical evaluation, surgical resection and adjuvant chemotherapy with imatinib mesylate is still the mainstay and most effective treatment for GISTs to date.

## HIGHLIGHTS

• Gastrointestinal stromal tumors are the most common mesenchymal tumor located in the gastrointestinal tract.
• The occurrence of gastrointestinal stromal tumors of the stomach presenting as an intratumoral abscess are extremely rare.
• Complete surgical resection is the only potential curative treatment for GISTs.
• Administration of Imatinib mesylate should be considered for these GISTs.

## 1 Introduction

Gastrointestinal stromal tumors (GIST) are the most common mesenchymal tumor located in the gastrointestinal (GI) tract. GIST are considered to develop from interstitial cells of Cajal (ICC), which play an important role in autonomous gastrointestinal movement. The most common localization of GIST is the stomach, followed by other gastrointestinal tract localizations [1]. GISTs of the stomach presenting as an intratumoral abscess are extremely rare, which necessitates emergency surgery, we report a case of a stomach GIST developing an intratumoral abscess, in whom emergency surgery was performed. The world-wide age-adjusted annual incidence rates range from 6.8 to 14.5 cases per million [2]. This work has been reported in line with the SCARE criteria [3].

## 2 Case presentation

**Fig. 1:**
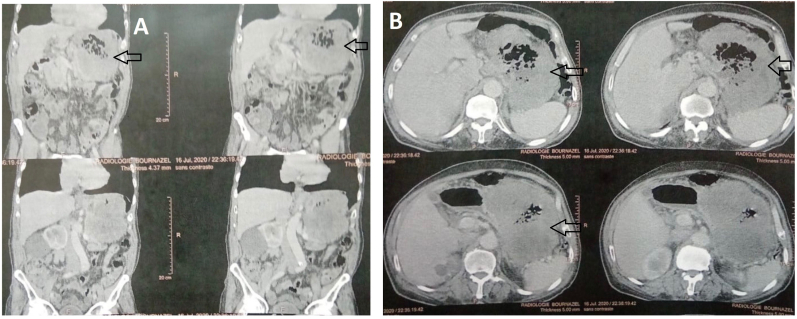
(A, B) Enhanced computed tomography (CT) scan of the abdomen (axial and coronal planes) revealing a large tumor (left arrow) adjacent to greater curvature of the stomach.

**Fig. 2:**
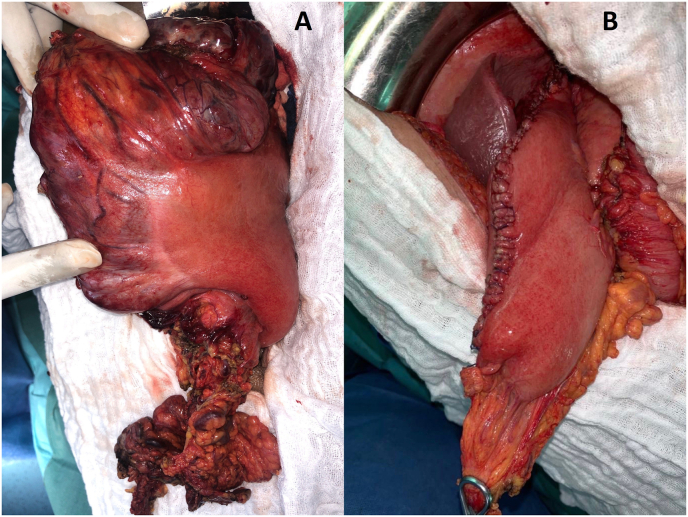
(A, B) Intraoperative photograph showing a tumor arising from the greater curvature before and after sleeve gastrectomy.

**Fig. 3:**
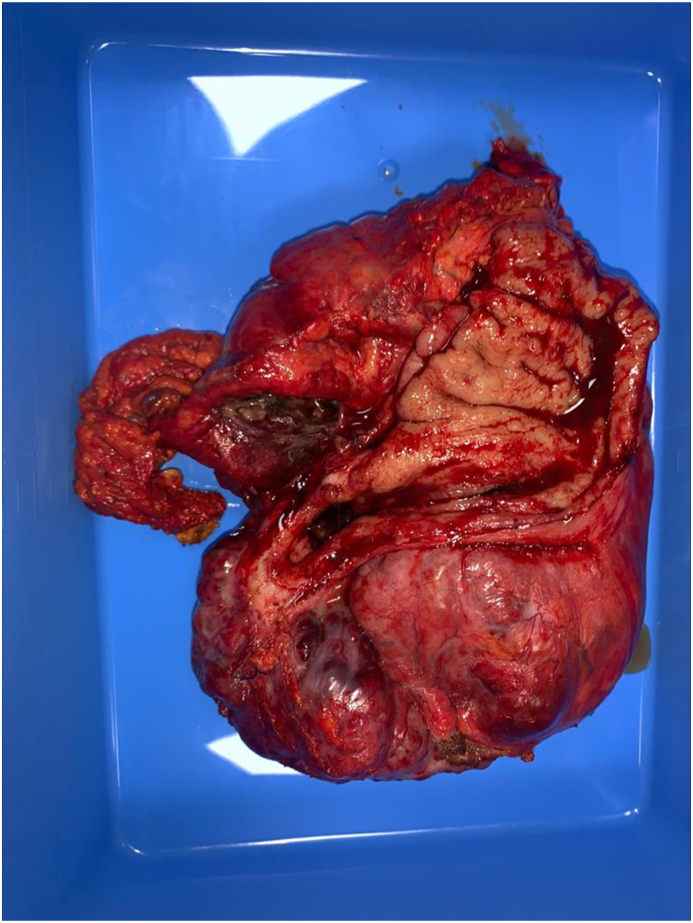
Specimen.

A 68-year-old man walked into the emergency room due to severe abdominal pain. The upper endoscopy with biopsy was performed 3 months ago and the diagnose of the GIST of stomach was revealed. Physical examination revealed fever of 39 °C blood pressure (88/50 mmHg) and heart rate (120 beats/min) and muscular defense around the upper abdomen. Laboratory data on admission showed white blood cell count of 30 250/mm3 and C-reactive protein of 340.1 mg/L. An enhanced computed tomography (CT) scan showed an irregularly 15 × 10 cm cystic mass adjacent to greater curvature of the stomach, which contained air (Fig. 1). No other visceral abnormalities were found. A combination therapy that includes systemic administration of antibiotics and an emergency laparotomy was performed. A giant cystic gastric mass was observed (Fig. 2). Sleeve gastrectomy were performed to excise the tumor (Fig. 3) and abscess. The histopathology of the gastric specimen showed a mass with 17 × 14 × 7 cm in size, without margin involvement (R0). The mass was diagnosed accordingly as GIST of gastric origin in high-risk category with immunohistochemical staining showed that the tumor cells were positive for c-KIT and CD34. The patient was discharged from hospital on the 11th postoperative day and has been treated with imatinib in the outpatient clinic without any medical problems.

## 3 Discussion

Gastrointestinal stromal tumors (GISTs) are specific mesenchymal tumors of the gastrointestinal (GI) tract and a majority of these tumors occur in the stomach (60%) and the small intestine (30%) [4]. GIST arises from the stomach, presented with abdominal pain, GI bleeding or palpable mass [5]. Cases with intratumoral abscess formation as in our case are also rare.

Histologically, they are of 3 types: spindle cell type (70%), epitheloid type (20%) and mixed type (10%). Mitotic index calculated by counting the number of mitoses per 50 high-power fields is an important indicator of proliferative activity and prognosis of GIST.

The availability of a KIT and PDGFRA tyrosine kinase inhibitor, imatinib mesylate, has dramatically changed treatment strategies for metastatic or unresectable GISTs [6].

Contrast-enhanced computed tomography (CT) is the most used and effective imaging modality of choice for detection of the primary tumor and neoplastic metastases, as well as monitoring of treatment response [7]. CT tumor characteristics such as size greater than 10 cm, calcifications, irregular margins, heterogeneous, lobulated, regional lymphadenopathy, ulceration, extraluminal and mesenteric fat infiltration are more likely to be associated with metastasis. Small volume intraperitoneal disease is often only detected on diagnostic laparoscopy and is responsible for the reported 10%–15% of false negative rate with dynamic CT. Magnetic resonance imaging (MRI) is an acceptable alternative and more accurate than CT for delineating rectal GISTs and in detecting liver metastasis, hemorrhage and necrosis. Positron emission tomography (PET) can be used for both initial evaluation and trending the disease's progression that may be useful for detecting unapparent metastases or an otherwise unknown primary site and determining the response to neoadjuvant targeted therapy. PET scans usually indicate tumor responsiveness to imatinib mesylate within days to weeks of induction therapy [2].

Esophagogastroduodenoscopy (EGD) with ultrasonography (EUS) is an essential diagnostic modality to acquire tissue for diagnosis, usually by fine needle aspiration (FNA) or core-needle biopsy. In addition, EUS is accurate in determining the depth of penetration and origin of these neoplasms and also allows one to potentially consider a hybrid endoscopy/laparoscopic resection [2]. In our case (CT) scan showed an irregularly 15 × 10 cm cystic mass adjacent to greater curvature of the stomach, which contained air.

Surgical resection is still the mainstay and most effective and the only potentially curative treatment when possible for GISTs to date. GISTs only require the achievement of R0 resection without violating the capsule of the mass, and lymphadenectomy is not necessary [8]. Laparoscopic surgery has the potential advantage of requiring smaller incisions and less bowel manipulation compared with open surgery [9]. In this particular case of gastric abscess an emergency Sleeve gastrectomy were performed to excise the tumor and abscess. Imatinib mesylate is effective as an adjuvant treatment following complete resection of gastric GIST, several large, retrospective reports suggest local recurrence rates as high as 40% and five-year survival rates as ranging between 40% and 90% [2,10,11]. Imatinib-use was associated with decreased recurrence rates [12]. The patient received adjuvant therapy based on imatinib.

## 4 Conclusion

There were few reports of an intratumoral abscess of the gastric GISTs, We present a rare case of a huge GIST of the stomach This GIST was large and accompanied with a closed intratumoral abscess in whom emergency surgery was necessary.

## Provenance and peer review

Not commissioned, externally peer-reviewed.

## Ethical approval

I declare on my honor that the ethical approval has been exempted by my establishment.

## Sources of funding for your research

None.

## Author contribution

Ahmed Ballati: Corresponding author writing the paper

Zakaria Essaidi and Layla El Attar: writing the paper

Driss Errguibi: study concept

Amal Hajri: writing the paper

Rachid Boufettal: study concept

Saad Rifki El Jai: correction of the paper

Farid Chehab: correction of the paper

## Registration of research studies

researchregistry2464

## Guarantor

DOCTEUR AHMED BALLATI

## Declaration of competing interest

The authors declare having no conflicts of interest for this article
